# The Inner Foreskin of Healthy Males at Risk of HIV Infection Harbors Epithelial CD4+ CCR5+ Cells and Has Features of an Inflamed Epidermal Barrier

**DOI:** 10.1371/journal.pone.0108954

**Published:** 2014-09-30

**Authors:** Maria P. Lemos, Javier R. Lama, Shelly T. Karuna, Youyi Fong, Silvia M. Montano, Carmela Ganoza, Raphael Gottardo, Jorge Sanchez, M. Juliana McElrath

**Affiliations:** 1 Vaccine and Infectious Disease Division, Fred Hutchinson Cancer Research Center, Seattle, Washington, United States of America; 2 Asociación Civil Impacta Salud y Educación, Lima, Perú; 3 US Naval Medical Research Unit 6 (NAMRU-6), Lima, Perú; 4 Department of Medicine, University of Washington, Seattle, Washington, United States of America; 5 Department of Laboratory Medicine, University of Washington, Seattle, Washington, United States of America; Karolinska Institutet, Sweden

## Abstract

Male circumcision provides partial protection against multiple sexually transmitted infections (STIs), including HIV, but the mechanisms are not fully understood. To examine potential vulnerabilities in foreskin epithelial structure, we used Wilcoxon paired tests adjusted using the false discovery rate method to compare inner and outer foreskin samples from 20 healthy, sexually active Peruvian males who have sex with males or transgender females, ages 21–29, at elevated risk of HIV infection. No evidence of epithelial microtrauma was identified, as assessed by keratinocyte activation, fibronectin deposition, or parakeratosis. However, multiple suprabasal tight junction differences were identified: 1) inner foreskin stratum corneum was thinner than outer (p = 0.035); 2) claudin 1 had extended membrane-bound localization throughout inner epidermis stratum spinosum (p = 0.035); 3) membrane-bound claudin 4 was absent from inner foreskin stratum granulosum (p = 0.035); and 4) occludin had increased membrane deposition in inner foreskin stratum granulosum (p = 0.042) versus outer. Together, this suggests subclinical inflammation and paracellular transport modifications to the inner foreskin. A setting of inflammation was further supported by inner foreskin epithelial explant cultures secreting higher levels of GM-CSF (p = 0.029), IP-10 (p = 0.035) and RANTES (p = 0.022) than outer foreskin, and also containing an increased density of CCR5+ and CD4+ CCR5+ cells (p = 0.022). Inner foreskin dermis also secreted more RANTES than outer (p = 0.036), and had increased density of CCR5+ cells (p = 0.022). In conclusion, subclinical changes to the inner foreskin of sexually active males may support an inflammatory state, with availability of target cells for HIV infection and modifications to epidermal barriers, potentially explaining the benefits of circumcision for STI prevention.

## Introduction

Multiple trials have demonstrated that male circumcision reduces the risk of infection by HIV-1 [1]–[3]. To explain this observation, one study postulated that intact foreskin adds vulnerable surface area for pathogen entry, as HIV infection rates correlate with foreskin size [4]. Other studies proposed that foreskin has increased target cells for HIV infection, such as CD4+ T cells, macrophages, and dendritic cells (DCs) [5]–[9].

The foreskin is a double-sided layer of stratified squamous epithelium, with keratinocytes replicating at the stratum basale (SB), differentiating at the stratum spinosum (SS), maturing at the stratum granulosum (SG) and degrading at the stratum corneum (SC). Undamaged epithelium contains a few dividing keratinocytes, and suprabasal layers of the skin express keratin 1,10 heterodimers, contributing to skin resistance, cornified envelope formation, and desmosomal/tight junction integrity [10], [11].

The foreskin may undergo microtrauma during sexual activity, with disrupted epithelial barriers permitting pathogen entry. In injured epithelium, fibronectin deposits at the SB to aid in synthesis of a new connective tissue matrix, and to provide substrate for keratinocyte migration to repair wounds [12]. Proximal to the injury, activated keratinocytes, expressing keratin 6, migrate to close the wound and provide resistance to mechanical stress [13]. SB keratinocytes also replicate rapidly to refill the wound [14]; the accelerated differentiation can cause parakeratosis, with nuclei intercalated in the SC. These classical markers of epithelial integrity have not been assessed in the foreskin of sexually active males, but could help determine if foreskin microtrauma is a factor in enhancing STI transmission.

Multiple studies have aimed to identify differences among foreskin anatomical sites, to test the hypothesis that the inner foreskin provides a permeable site for pathogen entry. HIV risk has been associated with sub-prepucial wetness [15], where the inner foreskin is in contact with the penile shaft. However, the epithelial structures associated with foreskin permeability have not been fully explored.

Most studies aiming to document the barrier functions of inner foreskin have focused on SC thickness. The SC is composed of consecutive filaments of cross-linked keratin 1, keratin 10, filaggrin, involucrin, cornified envelope proteins, and lipids, conferring strength, elasticity and protection [16]. SC thickness studies have provided contradicting results: one study reported thickening of inner foreskin relative to outer in men with a history of penile infections [17], two reported no differences [18], [19], and three documented thinning [9], [20], [21].

In addition to keratinocyte homeostasis and SC integrity, recent evidence indicates that skin permeability is further regulated by the structure of tight junctions (TJs) in the SG. Involucrin, a terminal marker of keratinocyte maturation, is synthesized in the SS and cross-linked in the SG to provide structural support for TJs [22]. TJ proteins, such as claudins and occludin, form homotypic interactions among adjacent cells and regulate paracellular transport of water, ions, and large molecules [23], [24]. Studies indicate that occludin and claudin 1 form a barrier for extracellular biotin in the healthy human SG, and leakage correlates with their disappearance from the cell membrane [25].

TJ proteins also respond to acute and chronic inflammatory signals that regulate trans-epithelial resistance [26]–[32]. Occludin is believed to play its mayor role in the leak transport pathway [31]–[33], which responds to inflammatory stimuli such as IFN-γ and TNFα by endocytosis of TJ components and regulates the passage of larger molecules, such as HIV and bacterial products in reconstructed monolayers [34]. The overexpression of occludin has been shown to increase sensitivity of the leak pathway in response to inflammatory cytokines [32], but its potential role in the entry of pathogens at the pluri-stratified foreskin is unknown.

To further examine reasons behind increased STI risk in uncircumcised men, we explored differences among skin factors in the inner and outer foreskin of sexually active men who have sex with men (MSM) at risk of HIV. We describe differences in the cornified envelope, in TJ proteins in the SG and SS, in secretion of inflammatory cytokines, and in the density of CCR5+ and CD4+ CCR5+ cells. Together our results indicate that these participants, with no clinical signs or symptoms of penile disease, have subclinical signs of chronic inflammation in the inner foreskin. This may facilitate pathogen entry and increase STI susceptibility in uncircumcised males.

## Materials and Methods

### Tissue Donors

We evaluated foreskin tissue samples collected in Lima, Peru during HVTN 914. They correspond to 20 HIV seronegative, 21–30 year-old MSM, who reported activities associated with high risk of HIV acquisition, such as inconsistent condom use and a median of 8 anal sex partners in the last 6 months. In Peru, the MSM population has an HIV seroprevalence that is a 40-fold higher than the general male population [35], and high-risk MSM experience a 2.2–3.5% HIV incidence rate [36], [37].

At the time of circumcision, no participant had signs or symptoms of genitourinary infections. Two were previously treated for syphilis infection and exhibited positive syphilis serology at low titers (Biomerieux RPR-nosticon-II, or Stanbio RPR-quicktest). Five were HSV-2 seropositive (Focus Diagnostics Herpeselect 2 ELISA IgG followed by western blot for indeterminate ELISA results) but had no visible lesions. One tested positive for *Chlamydia trachomatis* in urine PCR (APTIMA Combo 2 Assay). All participants were negative for *Neisseria gonorrhoeae* in urine PCR.

### Ethics Statement

At an initial screening visit, a counselor explained the study objectives to potential participants and obtained written informed consent for study participation. The informed consent discussion included risks and benefits to circumcision, as well as an emphasis upon the need to adhere to safety protocols before and after the procedure. A different study staff member conducted an assessment of understanding and clarified any misconceptions of the key concepts discussed during the study consenting process. The study protocol, informed consent documents, and recruitment materials were approved by Institutional Bioethics Committee/Review Boards from Asociacion Civil Impacta Salud y Education, US Naval Medical Research Unit No. 6, and the Fred Hutchinson Cancer Research Center.

### Computer-assisted self interviewing (CASI) questionnaire

Oral, vaginal and anal sex behavior (receptive and insertive) was surveyed with a self-administered, adaptive, 35-question assessment, which was previously evaluated for terminology and cultural competence through five in-depth interviews and two focus groups of MSM in Lima, Peru.

### Foreskin Fixation, Embedding and Staining for Microscopy

Foreskin tissue was removed into 1× PBS with penicillin, streptomycin, and Fungizone (Life Technologies) and transported on ice. Within 2 h, tissues were dissected into inner and outer foreskin, sectioned into 5 mm×2.5 mm pieces, and fixed in buffered formalin for a maximum of 7 d. They were then dehydrated in 70% ethanol, paraffin embedded, and blinded regarding inner and outer status. Sections of 4 µm were stained for H&E or immunofluorescence microscopy.

Antigen retrieval for claudin 4 and Ki-67 was a 40 min 97°C incubation in Target Retrieval Solution pH 6.0 (DakoCytomation). Antigen retrieval for claudin 1, occludin, CD4, and CCR5 was a 20 min 97°C incubation in EDTA pH 8 (Trilogy; Cell Marque). Slides were then placed in TBS (Fisher) with 0.1% Tween-20 (Sigma) and loaded onto the DakoCytomation Autostainer.

For TJ stains, a convenience sample of the first 11 participants who underwent circumcision in HVTN 914 were selected to fit in a single run of the DakoCytomation Autostainer. Blocking started with an 8 min incubation of 3% H_2_O_2_ (VWR); this was omitted for the CD4 CCR5 stain because it affected CD4 staining. Then, all slides were blocked using the Biotin Blocking System as directed (Biocare) and a 10 min incubation with serum block. Claudin 4 and Ki-67 stains used 15% goat serum and 5% human serum diluted in TBS, 0.1% Tween-20, and 1% bovine serum (Sigma). Claudin 1 and occludin stains used Serum-free Protein Block (Dako). CD4 and CCR5 stains used 15% goat serum and 5% human serum diluted in Serum-free Protein Block.

Primary antibodies and isotype controls were diluted in TBS, 0.1% Tween-20, and 1% bovine serum and applied at equivalent concentrations for 60 min at room temperature; developing reagents were applied for 30 min. Anti-Ki-67 (MIB1, Dako) and mouse IgG (Vector) were followed by biotin-conjugated goat anti-mouse IgG1 F(ab′)2 (Jackson Immunoresearch) and streptavidin-AlexaFluor350 (Life Tech). Anti-Claudin 4 (Novus) and -Claudin 1 (Thermo) were developed with goat anti-rabbit AlexaFluor647 (Life Tech). Anti-occludin (Sigma) and rabbit IgG (Jackson) were developed with goat anti-rabbit AlexaFluor647, amplified using a Cy5 antibody (Sigma), and followed by goat anti-mouse AlexaFluor647 (Life Tech). Anti-CD4 (SP35, Cell Marque) and rabbit IgG were followed by biotin-conjugated goat anti-rabbit IgG (Dako) and amplified using Dako CSA/ABC Amplification Reagent, and streptavidin-AlexaFluor350. Anti-CCR5 (provided by M. Mack, University Hospital Regensburg, Germany) and mouse IgG (Jackson) were developed with goat anti-mouse AlexaFluor647. All slides were counterstained with SYTOX Orange (Life Tech), coverslipped with ProLong Gold (Life Tech), cured and stored at 4°C.

Antibody staining procedures were validated by testing each antibody specificity in at least three human tissues: intestinal biopsies, abdominal skin and lymph node biopsies, which corresponded to the staining presented in the human protein atlas (http://www.proteinatlas.org). They were all validated by a trained pathologist.

### Imaging and Microscopy Analysis

Slides were imaged using a semi-automated digital pathology TissueFAXS system (Tissuegnostics GmbH) containing a Zeiss Imager Z2 upright microscope (Carl Zeiss Microscopy) with an 8-slide motorized stage (Marzhauser Wetzlar GmbH). A Plan Apochromat 20×/0.8 objective was used to take individual images, and composite images for the entire section were compressed 50%.

Transmitted light images of H&E slides were acquired by a PL-B622CF color CCD camera (Pixelink). To incorporate potential spatial variability within distal and proximal parts of the foreskin, median thickness is a summary measurement of 3–5 inner or outer foreskin sections covering ∼26.5 mm^2^ (interquartile range [IQR], 19.5–34.8) and extending at 5 mm intervals from the foreskin tip to the base of the shaft. A composite image of all the fields of view at 10× magnification was used for analysis. Using ImageJ (NIH), the epithelium or SC area was manually outlined in each section, blinded to participant and foreskin location. On the H&E slides, the epithelium had hematoxylin staining keratinocytes, and the SC was clearly identified as the external, eosinophilic, acelullar layer. To determine average thickness in each section, outline area measurements were divided by Feret's diameter of the outline, which closely approximates the length of the epidermis or SC in each section. Parakeratosis was measured by counting nuclei in the SC of 3–5 sections from inner and outer foreskin. To estimate nuclei density, the nucleus count was divided by the SC Feret's diameter. All length measures were converted to micrometers using objective magnification and image compression.

Fluorescence images were acquired on a Pixelfly QE monochrome CCD camera (PCO Imaging) using filter cubes 330–380 nm Ex/435–485 nm Em (Blue), 540–580 nm Ex/592–667 nm Em (Red), and 590–650 nm Ex/673–762 nm Em (Far Red). Composite images covering a median tissue area of ∼26.5 mm^2^ were analyzed using Cell Profiler (Broad Institute), an open-source software to quantitatively measure phenotypes from thousands of images automatically using identical settings [38], [39].

Autofluorescence of the epidermal layer (from the SB to the SC) on the red channel was used to outline the epidermis area. Cell Profiler's thresholding methods were used to define which pixels were attributed to background and foreground intensities. Total nuclei were determined by counting SYTOX orange stained nuclei within the epidermis. Proliferating keratinocytes were determined by Ki-67+ staining after subtracting each section's isotype control staining (median 0.17%).

The percentage of epidermal area covered by a TJ stain was calculated after subtracting isotype control staining (ranging from 0.02% to 0.06%). Mean intensity of TJ stains within the epidermis was calculated by adding the fluorescence from each pixel in the epidermis (scaled in arbitrary units ranging from 0 [lowest] to 1 [highest]) and dividing by the epidermal area of each foreskin section. Background isotype control staining was subtracted (median 1.3% intensity for rabbit IgG [claudin 1], 2.5% for mouse IgG [occludin], 2% for rabbit IgG [claudin 4]). Serial H&E sections cut in parallel with the immunofluorescent stained slides were used to provide morphological characterization of the SG, as keratohyalin granules stain with hematoxylin.

The polarization of membrane claudin 4 staining was calculated using the angular second moment, where the square of each epidermal pixel's fluorescence (expressed in arbitrary units [0–1]) was multiplied by its radial distance from the epidermal midpoint. It serves as a measure of the nonuniformity of the fluorescence distribution in an area, and provides evidence of structural variations [40], such as the accumulation of fluorescent intensity in the SC, away from the SB.

For measurement of CD4+ and CCR5+ cells, each pixel's fluorescence in the blue (CD4) and far red (CCR5) channels was expressed in arbitrary units (0–1) and cells were identified as 10–33 µm^2^ areas with aggregate intensity above background threshold (0.03 for CD4, 0.025 for CCR5). Background isotype control staining for each subject was subtracted (median 4.2 cells/mm^2^ epidermis for mouse IgG [CCR5], 2.1 cells/mm^2^ for rabbit IgG [CD4]).

### Foreskin Lysate Preparation

Inner and outer foreskin pieces, 5 mm×2.5 mm, were taken 1 cm from the foreskin tip and frozen at −80°C within 2 h of circumcision. Tissue was homogenized with a wide probe homogenizer (Omni) in PBS with 0.5% propylene glycol (Sigma), 0.2% SDS (Sigma), and 20 µl/ml Protease inhibitor cocktail Set I (Millipore) [41]. Lysate protein concentrations were measured by bicinchoninic acid assay kit (Pierce) and normalized to 200 µg protein/ml.

### Foreskin Explant Cultures

Within 2 h of collection, 5 mm×2.5 mm pieces of inner or outer foreskin (extending from the base of the penis to the tip) were cultured for 2 h in 1× PBS with 4 U/ml dispase (Worthington). Epidermal sheets were separated from dermis and washed extensively in PBS, then cultured independently in RPMI 1640 with 20% Benchmark FBS (Gemini), penicillin, streptomycin, fungizone, and L-glutamine (Life Technologies). After 48 h, debris was removed by 300×*g* centrifugation, and supernatants were collected and stored at −80°C until assessment. As only the dermis is vascularized, hemoglobin ELISA (ICL) was used to assess dermal contamination in epidermal explant cultures. Epidermal cultures with hemoglobin concentrations ≥1 confidence interval above the assay limit of detection were excluded from analysis.

### Multiplex Bead Array (MBA)

Human Cytokine and Human Skin Panel multiplex assays were assessed in a Luminex platform using manufacturer's instructions (Millipore). Lysate buffer was used for background measurements in the skin panel; RPMI with 20% FBS was used for the cytokine panel. Curve fitting for MBA standards used the nCal R package [42]. Extrapolated data from the MBA was analyzed using the ratio of inner versus outer analyte concentrations. Global differences among the inner/outer ratios of explant culture cytokines or protein lysates were explored using the Wilcoxon signed ranked test to determine if each analyte ratio was different from 1.

### Statistical Analysis

Statistical comparisons were carried out using Prism v6 (GraphPad Software). Unblinding of the inner/outer status was carried out after collection of experimental data. All comparisons between inner and outer foreskin measurements were evaluated using Wilcoxon matched-paired signed ranked tests. To account for multiple comparisons, all p values were adjusted using the Benjamini-Hochberg procedure [43] for 53 comparisons. A false discovery rate (FDR) corrected p<0.05 was considered significant, and p<0.10 was considered a trend.

## Results

### Keratinocyte turnover and basal skin homeostasis are comparable between inner and outer foreskin mucosa from sexually active young males

To examine the homeostasis of foreskin epithelium layers, we studied foreskins collected following elective circumcision of 20 healthy, sexually active MSM, ages 21–29, at risk of HIV infection, but with no signs or symptoms of penile disease. To investigate any evidence of foreskin microtrauma associated with sexual activity, we compared inner and outer foreskin from the 17 participants who reported insertive sex or penile masturbation in the two weeks prior to circumcision, because epithelium turnover takes ∼14 d [44] and recent sexual activity could have left evidence of epithelial damage.

**Figure 1:**
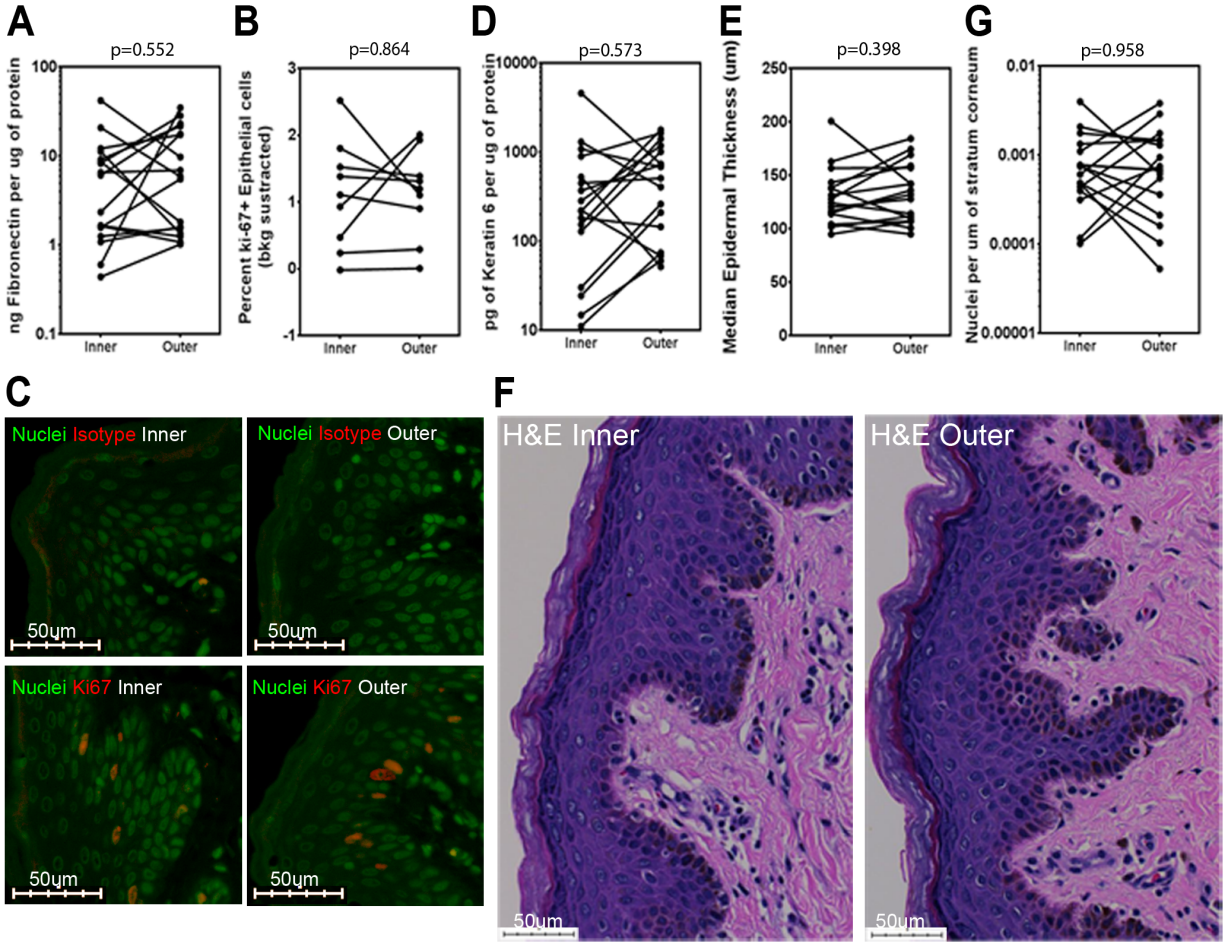
No differences in keratinocyte turnover and skin homeostasis among inner and outer foreskin mucosa from sexually active men. A) Fibronectin and D) keratin 6 concentrations in foreskin lysates from all subjects sexually active within the past 2 weeks (n = 17) were measured by MBA. B) Percent of Ki-67+ nuclei among all epithelial nuclei on sections from 9 participants. C) Representative staining for Ki67 and SYTOX orange (nuclei). E) Median epithelial thickness (µm) was computed measuring 3–5 H&E-stained sections of ∼26.5 mm^2^ inner/outer foreskin for each participant (n = 17). F) Representative H&E staining G) Parakeratosis was quantitated from H&E sections from all subjects (n = 17). All p values are from FDR-adjusted Wilcoxon tests.

We first compared basal epithelium repair and microtrauma between inner and outer foreskin, but found no differences in fibronectin as assessed by MBA (p = 0.552; Figure 1A), or in the fraction of proliferating and activated epidermal keratinocytes by immunofluorescence microscopy (p = 0.864; Figure 1B and 1C). Additionally, we saw no differences in keratinocyte activation in the suprabasal layers, based on keratin 6 expression by MBA (p = 0.573; Figure 1D). Epidermal thickness among inner and outer foreskin tissues was also comparable in H&E stained sections (p = 0.398; Figure 1E and 1F). Lastly, parakeratosis was rare and showed no significant differences (Figure 1F and 1G; p = 0.958). Similar results were observed when the studies were carried out in the entire cohort, regardless of recent sexual activity (data not shown).Together, the results indicated no evidence for increased repair or microtrauma in the inner foreskin epidermis of sexually active males at risk of HIV infection.

### Healthy, sexually active, young males have thinning of inner foreskin SC

**Figure 2:**
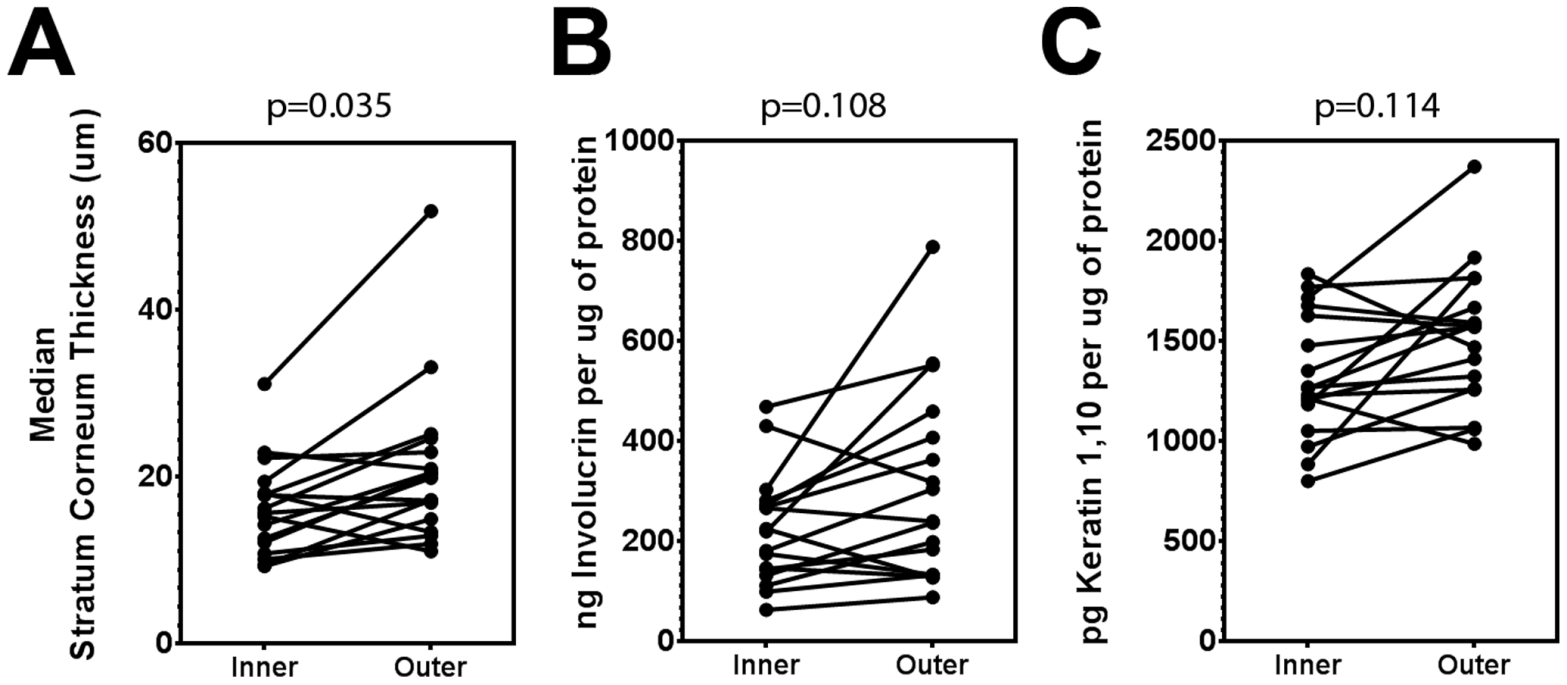
Sexually active MSM have a slightly reduced SC envelope in the inner foreskin. A) Median SC thickness (µm) was computed from 3–5 sections of inner/outer foreskin, each taken 5 mm apart. B) Involucrin and C) keratin 1,10 levels were measured by MBA in foreskin lysates. All p values are from FDR-adjusted Wilcoxon comparisons of all subjects sexually active within the past 2 weeks (n = 17).

To further study barrier functions in the inner and outer foreskin SC, H&E stained longitudinal sections were analyzed for SC thickness in the 17 sexually active participants. The inner foreskin SC (median, 15.69 µm; IQR, 11.50–18.76) was significantly thinner than the outer (median, 19.90 µm; IQR, 14.19–23.83; p = 0.035; Figure 2A). To confirm these SC thickness findings, levels of involucrin and keratin 1,10 dimers were measured by MBA in these subjects; but the differences were not significant after FDR adjustment (Figure 2B and 2C). Similar results were observed when the studies were carried out in the entire cohort, regardless of recent sexual activity (data not shown).Together, the trends suggest there is thinning of the inner foreskin in this population, when compared to the outer foreskin.

### The inner foreskin SG and SS has modified TJ localization

**Figure 3:**
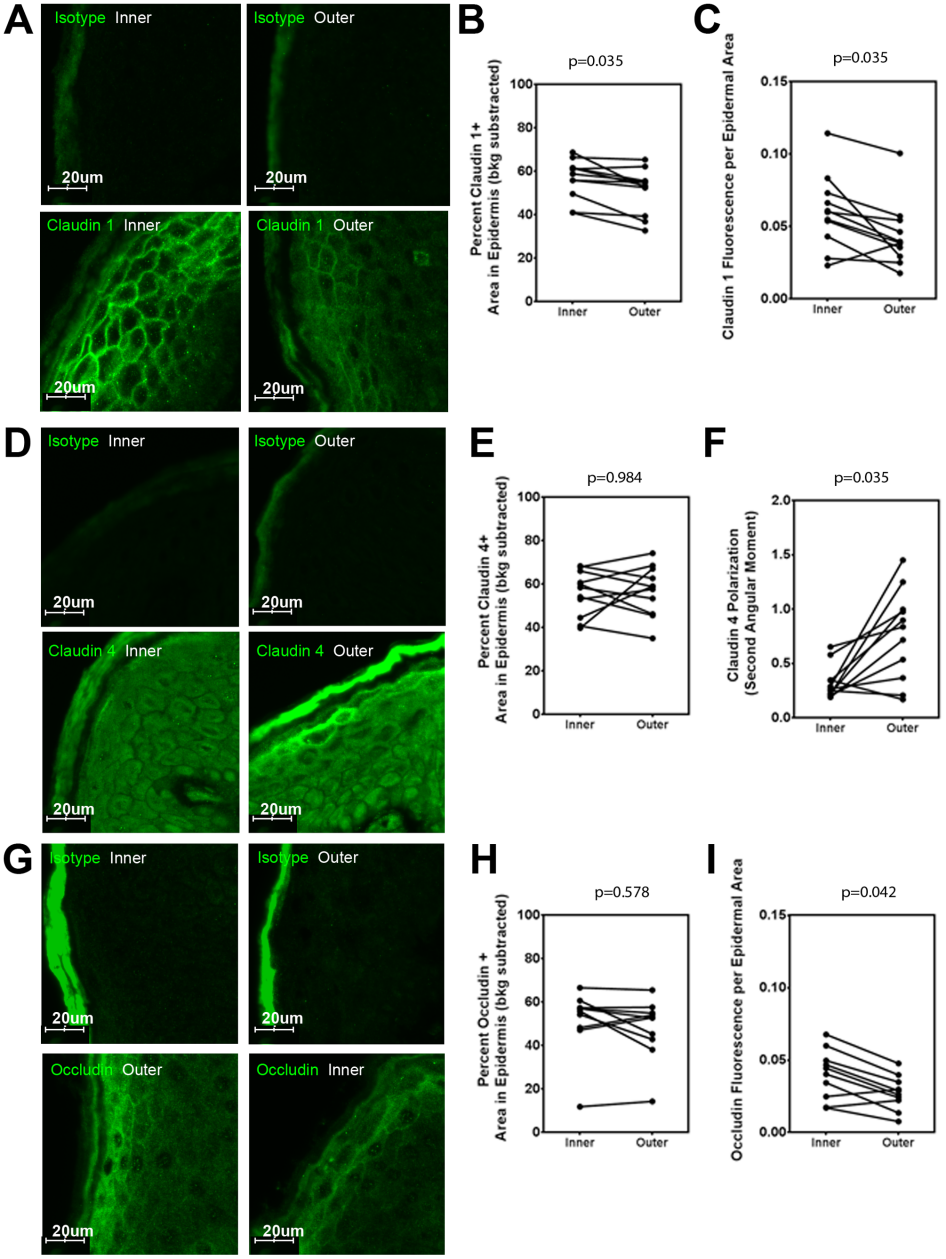
Tight junction proteins differentially accumulate in the inner and outer foreskin sections from sexually active men. Samples from 11 participants were selected for immunofluorescence studies. A, D, G) Representative images of foreskin epidermis at 40× magnification stained with A) claudin 1, D) claudin 4, and G) occludin (pseudocolored green) or their isotype controls. All the fields in ∼26.5 mm^2^ of tissue per participant were used for analysis. B, E, H) Percent of epidermal area covered by B) claudin 1, E) claudin 4 or H) occludin stain. C, I) Mean intensity of C) claudin 1 or I) occludin within the epidermis. F) The angular second moment of claudin 4 staining within foreskin epidermis. All p-values are from FDR-adjusted Wilcoxon tests.

Since TJs also contribute to skin paracellular permeability, we explored their integrity using immunofluorescence microscopy. Paraffin-embedded sections of inner and outer foreskin from 11 males were stained for claudin 1, claudin 4, and occludin (Figure 3).

Claudin 1 accumulated in the membranes of 3–5 layers of terminally differentiated keratinocytes at the SG and SS of outer foreskin (Figure 3A). In contrast, it was more broadly localized in inner foreskin; membrane-bound claudin 1 extended throughout most suprabasal layers, sometimes reaching just above the SB. The area of epithelial claudin 1 staining within inner foreskin (median, 58.85%; IQR, 49.67–61.59) was slightly larger than outer foreskin (median, 53.27%; IQR 39.38–55.59; p = 0.035; Figure 3B). The average staining intensity of claudin 1 was also greater in inner foreskin epithelium (median, 0.060 AU/pixel; IQR, 0.043–0.073) versus outer (median, 0.039 AU/pixel; IQR, 0.029–0.054; p = 0.035; Figure 3C), indicating increased membrane localization.

Claudin 4 staining was less intense than claudin 1 and mainly cytosolic (Figure 3D). In contrast to skin, both inner (median, 58.45%; IQR, 44.55–66.06) and outer foreskin (median, 58.86%; IQR, 46.22–67.05) had intracellular claudin 4 staining throughout the suprabasal epidermis and there were no differences in the epidermal area covered (Figure 3E, p = 0.984). There were also no differences in average staining intensity in inner foreskin (median, 0.038 AU/pixel; IQR, 0.027–0.060) versus outer (median, 0.047 AU/pixel; IQR, 0.038–0.059; p = 0.349).

However, a membrane-bound layer of claudin 4 at the SG was only seen within outer foreskin, as indicated by the polarization of staining intensity towards the distal epithelial layers (Figure 3D and 3F). Claudin 4 polarization was higher towards the SC in outer foreskin epidermis (median, 0.838 AU/pixel; IQR, 0.367–0.998) when compared to inner (median, 0.267 AU/pixel; IQR, 0.218–0.348; p = 0.035), which lacked membrane-bound claudin 4. This suggested that inner and outer foreskin express different membrane-bound claudins, and may differentially regulate water and small solute transport.

Occludin showed areas of high intensity staining in keratinocyte membranes under the SC of both inner and outer foreskin (Figure 3G). Intracellular staining was detected throughout the epithelial surface (Figure 3G and 3H) and no significant differences in epithelium coverage were observed among inner (median, 56.42%; IQR, 47.97–58.18) and outer foreskin (median, 52.77%; IQR, 41.67–55.61; p = 0.578). However, inner foreskin had increased staining intensity (median, 0.042 AU/pixel; IQR, 0.023–0.052) versus outer (median, 0.027 AU/pixel; IQR, 0.020–0.036; p = 0.042; Figure 3I). Together, these results indicated there are TJ modifications in the inner foreskin SS and SG in this population.

### Inner foreskin epithelium can secrete increased levels of inflammatory cytokines

**Figure 4:**
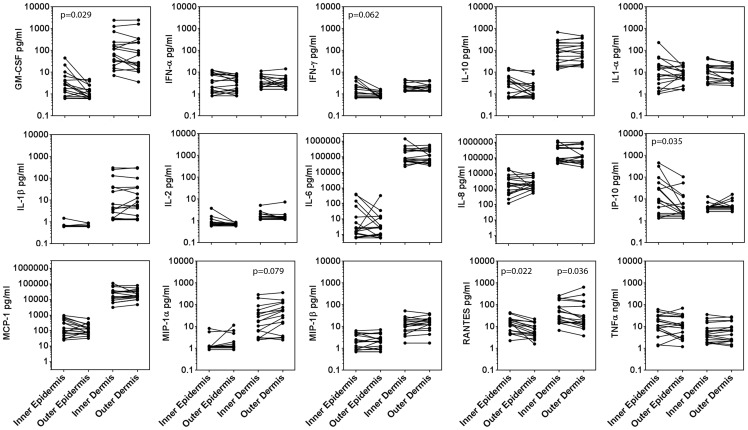
Both the inner foreskin epidermis and dermis from sexually active MSM can secrete increased levels of inflammatory cytokines compared to the outer foreskin. Supernatants from explant cultures from participants who were sexually active within the past 2 weeks (n = 17) were quantitated for GM-CSF, IFN-α, IFN-γ, IL-10, IL-1α, IL-1β, IL-2, IL-6, IL-8, IP-10, MCP-1, MIP1α, MIP1β, RANTES and TNFα using MBA to measure cytokines in dermal and epidermal explants after 48 h culture. All p values are from FDR-adjusted Wilcoxon tests.

We compared secretion of GM-CSF, IFN-α, IFN-γ, IL-10, IL-1α, IL-1β, IL-2, IL-6, IL-8, IP-10, MCP-1, MIP1α, MIP1β, RANTES, and TNFα from dermal and epidermal explants after 48 h culture to determine if soluble indicators of inflammation were more prevalent in inner foreskin epidermis (Figure 4). Three individuals were excluded from analysis because hemoglobin was detected in their non-vascularized epidermal explants, indicating dermal contamination.

Among the remaining 17 individuals, the inner foreskin epidermis among had increased concentrations of GM-CSF, IFN-γ, IP-10, and RANTES. Inner foreskin secretion of IP-10 (median, 9.9 ng/ml; IQR, 2.0–44.0) was four-fold higher than outer (median, 2.2 ng/ml; IQR, 1.9–9.7; p = 0.035). Inner foreskin secretion of GM-CSF (median, 2.7 ng/ml; IQR, 0.8–5.5) was three-fold higher compared to outer (median, 0.94 ng/ml; IQR, 0.7–2.1; p = 0.029). Inner foreskin secretion of RANTES (median, 14.0 ng/ml; IQR, 5.8–21.2) was two-fold higher than outer (median, 5.7 ng/ml; IQR, 3.9–13.2; p = 0.022). Lastly, inner foreskin secretion of IFN-γ (median, 1.0 ng/ml; IQR, 0.7–2.3) trended 1.25 fold higher than outer (median, 0.88 ng/ml; IQR, 0.7–1.0; p = 0.062), but this difference was not significant after FDR adjustment.

We also compared inner and outer dermal cytokine secretion from all study participants and found trends of differential secretion of RANTES and MIP-1α (Figure 4). MIP-1α differences were not significant after FDR adjustment (p = 0.079), but inner foreskin secretion of RANTES (median, 30.0 ng/ml; IQR, 16.1–124.5) was 1.5 fold higher than outer (median, 19.8 ng/ml; IQR, 12.3–113.1; p = 0.036). Together, the results indicate that inflammatory cytokines may concentrate immune cells in the inner foreskin.

### The inner foreskin epithelium contains increased numbers of HIV target cells

**Figure 5:**
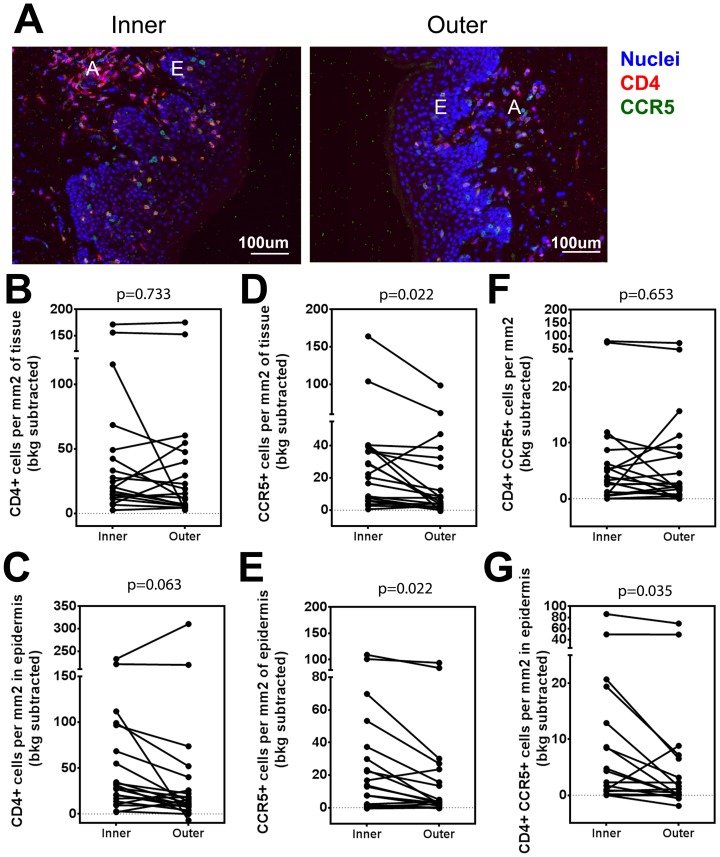
CCR5+ cells accumulate in the inner foreskin in from sexually active MSM. A) Representative images of foreskin epidermis at 10× magnification stained with CCR5 (pseudocolored green), CD4 (pseudocolored red) and SYTOX Orange (pseudocolored blue) for nuclear identification. All fields from ∼26.5 mm^2^ sections were used for analysis. For isotype controls and larger tissue sections see **Figure S1**. White letters mark CD4+ cell aggregates (A), and epidermis (E). B–G) Cell density measured in the B, D, F) whole tissue (dermis and epidermis) or C, E, G) epidermis. Cell types measured are B, C) CD4+, D, E) CCR5+ and F, G) CD4+ CCR5+ cells. All p-values are from FDR-adjusted Wilcoxon tests.

A pathologist review of H&E slides identified dermal lymphocytic aggregates in both inner and outer foreskin in these participants (data not shown). We explored whether the signs of inflammation in the inner foreskin are associated with increased density of CD4+ CCR5+ cells. Paraffin-embedded sections of inner and outer foreskin, collected 1 cm from the glans tip from all participants (n = 20), were stained for SYTOX Orange (nuclei), CD4 and CCR5 (Figure 5A and S1). CD4+ cells and CCR5+ cells formed aggregates in the dermis of both inner and outer foreskin (Figure 5A, letter A) and scattered cells also infiltrated the epidermis (Figure 5A, letter E). We also detected no significant differences in whole tissue CD4+ cell density among inner and outer foreskin (p = 0.733; Figure 5B), but saw an increased trend of CD4+ cells in the inner foreskin epidermis (median 29.94 cells/mm^2^, IQR 14.49–89.73) versus outer epidermis (median 14.5 cells/mm^2^, IQR 7.78–36.56; p = 0.0637; Figure 5C).

Interestingly, there was a higher density of CCR5+ cells in the inner foreskin (median 21.34 cells/mm^2^, IQR 6.36–38.64) versus outer (median 6.46 cells/mm^2^, IQR 1.98–31.04) when whole tissue sections were examined (p = 0.022; Figure 5D). Similar results were observed when only the epidermal area was compared (inner median, 13.30 cells/mm^2^, IQR 1.59–35.42; outer median, 3.21 cells/mm^2^, IQR 0.27–21.54; p = 0.022; Figure 5E). This finding suggests preferential CCR5+ cell presence at the inner foreskin.

Lastly, we explored the accumulation of CD4+ CCR5+ cells in the inner and outer foreskin. No differences were identified when whole tissue sections were analyzed (p = 0.653; Figure 5F). But when the epidermis was examined independently, we observed more CD4+ CCR5+ cells in inner foreskin (median 2.01 cells/mm^2^, IQR 0.04–11.83) compared to outer (median 0.25 cells/mm^2^, IQR 0.00–5.68; p = 0.035; Figure 5G), indicating presence of potential HIV target cells to the area. This finding was consistent with other signs of inflammation and supported the idea that chronic inflammation of the inner foreskin epidermis is occurring *in vivo*.

## Discussion

The epithelium serves as the primary barrier to many infectious agents, including STIs. Circumcision provides partial protection against many STIs, but the reasons for this remain unclear. We reasoned that the foreskin fold creates two different environments for the genital skin: one represented by the inner foreskin, which can be humid [15] and capable of harboring anaerobic bacteria [45]; another represented by the outer foreskin which is exposed to air. In comparing these two regions, we found differences in the inflammatory milieu of inner versus outer foreskin, as well as epithelial modifications that could affect permeability and thus susceptibility to infection.

### Markers of chronic suprabasal inflammation in the inner foreskin epidermis

Although we observed no differences in keratinocyte damage, activation, replication, or fibronectin deposition, and rare dermal pathology, the TJ distribution is reminiscent of inflammatory settings. Uninvolved psoriatic skin has been shown to overexpress claudin 1 [28] in an attempt to compensate early for inflammatory cascades in the epithelium before the initiation of local symptoms. Exposure of epithelial monolayers to IFN-γ can drive the compensatory accumulation of claudin 1 into membrane-bound compartments [46].

Expression of claudin 4 protein is normally restricted to the SG; but it disappears from this location in psoriatic plaques [29], [30].These reports resemble the TJ conformation that we see in the inner foreskin of sexually active males, suggesting this genital site might have adapted to subclinical, chronic inflammation.

Occludin deposition in the inner foreskin may contribute to link inflammation to TJ remodeling, as it mediates the cytokine-mediated breakdown of TJs through the leak pathway, which allows for large molecule paracellular transport [31]–[33]. A recent study demonstrated that foreskin explants cultured with exogenous TNFα activated inner but not outer foreskin DCs [47], suggesting that TNFα, the major leak pathway trigger [31]–[33], has differential access to inner vs outer foreskin. Thus, in the foreskin, the epidermal barrier and the immune system may play enhancing roles in initiating and sustaining TJ modifications, like they do in many skin diseases [16].

The presence of an inflammatory milieu in the foreskin is further supported by the increased secretion of GM-CSF, IP-10, and RANTES from inner foreskin epidermal explants, as well as increased density of CCR5+ and CD4+ CCR5+ cells to inner epidermal areas. Although other studies indicated that foreskin has HIV target cells [5]–[8], our study contributes to the published studies by providing evidence that CD4+ CCR5+ cells reside in the stratified epithelium of the inner but not outer foreskin of sexually active MSM. This is consistent with an explant model showing that elevated RANTES contributes to foreskin epidermal migration of CD3+ T cells and facilitates cell-associated HIV transmission in the inner foreskin [48]. Thus, the inflammatory milieu of inner foreskin may help position HIV target cells in a region where the epidermis could potentially facilitate both HIV entry and infection.

However, our study cannot distinguish whether these inflammatory features represent the natural state of the male foreskin, or whether they are unique to our study population. In support of the first hypothesis, recent reports suggest uncircumcised men have increased representation of anaerobic bacterial families in their coronal sulcus when compared to circumcised men [45], and circumcision reduces this anaerobic bacterial load [49]. Bacterial families overrepresented in the penile skin of uncircumcised men might trigger TJ alterations and inflammatory cell recruitment. Acute exposure to *Staphylococcus* sp. or *Clostridium* enterotoxin, for example, can relocalize TJ proteins away from the plasma membrane and decrease trans-epithelial resistance [26], [27]. In support of the second hypothesis, anal intercourse is associated with subprepucial wetness [50], and rectal anaerobic bacteria could colonize penile surfaces, providing a unique permeable, inflammatory environment.

### Potential consequences for the permeability in the inner foreskin

Our data indicate the inner foreskin tissue from sexually active young males at risk of HIV has 20% less SC thickness than the outer. This difference, if significant for permeability, is small, which may explain the mixed results reported by others [9], [17]–[21]. The differences among studies might also represent distinct study populations, as other studies were not able to control for participant age, lacked information about STI history and sexual activity, and recruited participants from other continents.

In our relatively small cohort, our SC measurement approach has the advantage of incorporating measurements from large skin areas and encompassing data from different regions of inner and outer foreskin. Even so, we failed to identify significant reductions in cornified envelope proteins after FDR adjustment. This might be attributed to the differences being quite small, or may imply that inner foreskin thickness reflects modifications in other components, such as water, lipids, or other less dominant SC proteins.

Our study also indicates the inner foreskin of males at risk of infection has TJ alterations such as the absence of membrane-bound claudin 4, extended membrane localization of claudin 1, and a more intense occludin staining pattern, suggestive of permeability differences. Although apparently small in magnitude, it is notable that modifications to the substrata were significant enough to affect our total epidermal summary measures. However, it will be important to test the permeability of the foreskin regions experimentally, to establish whether the SC and SG modifications could allow for differential bacterial and viral translocation.

### Conclusion

In summary, the inner foreskin of sexually active males at risk of HIV infection has a thinner SC and modifications to the TJs, which are barriers against pathogens. Inflammatory modifications to TJs of the inner foreskin SG are consistent with increased secretion of cytokines and higher densities of CCR5+ and CD4+ CCR5+ cells in the inner foreskin epithelium. Thus, the combination of increased inflammation and alterations to epithelial barriers at the inner foreskin may contribute to STI susceptibility, especially HIV, in uncircumcised men.

## Supporting Information

Figure S1 **Representative images of foreskin epidermis at 10× magnification stained with CCR5 (pseudocolored green), CD4 (pseudocolored red) and SYTOX Orange (pseudocolored blue) for nuclear identification and their respective isotype controls.** White letters mark CD4+ cell aggregates (A), and epidermis (E).
(TIF)
