# Supplementary figures and images for: The Inner Foreskin of Healthy Males at Risk of HIV Infection Harbors Epithelial CD4+ CCR5+ Cells and Has Features of an Inflamed Epidermal Barrier
Source: PLoS One. 2014 Sep 30;9(9):e108954. doi: 10.1371/journal.pone.0108954 (PMC4182607; doi:10.1371/journal.pone.0108954)

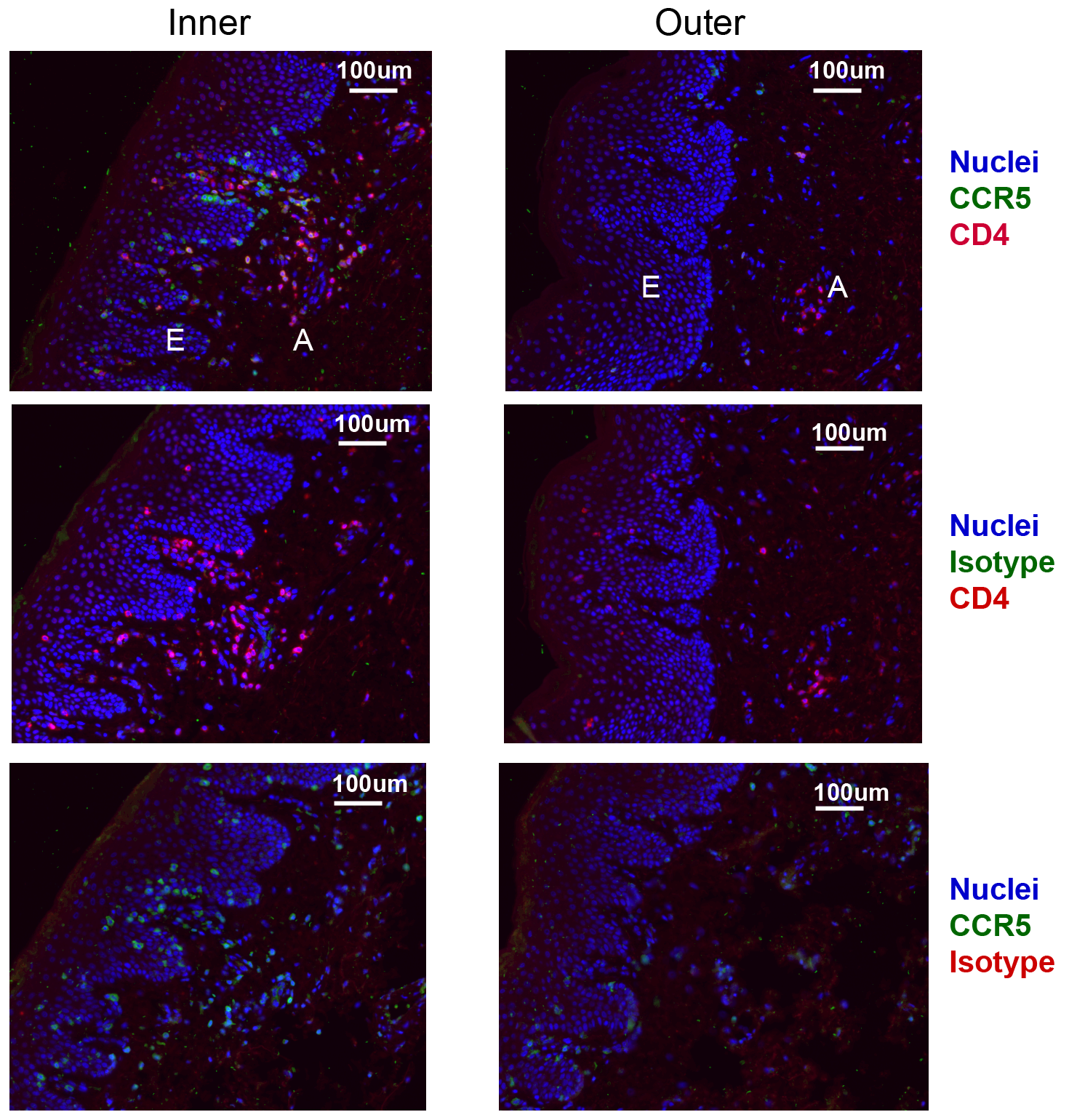

Supplement: Figure S1 — Representative images of foreskin epidermis at 10× magnification stained with CCR5 (pseudocolored green), CD4 (pseudocolored red) and SYTOX Orange (pseudocolored blue) for nuclear identification and their respective isotype controls. White letters mark CD4+ cell aggregates (A), and epidermis (E). (TIF) [file pone.0108954.s001.tif]
